# TM4SF5-Mediated Roles in the Development of Fibrotic Phenotypes

**DOI:** 10.1155/2017/5108525

**Published:** 2017-03-26

**Authors:** Jihye Ryu, Jung Weon Lee

**Affiliations:** Department of Pharmacy, Research Institute of Pharmaceutical Sciences, Tumor Microenvironment Global Core Research Center, Medicinal Bioconvergence Research Center, College of Pharmacy, Seoul National University, Seoul 08826, Republic of Korea

## Abstract

Transmembrane 4 L six family member 5 (TM4SF5) can form tetraspanin-enriched microdomains (TERMs) on the cell's surface. TERMs contain protein-protein complexes comprised of tetraspanins, growth factor receptors, and integrins. These complexes regulate communication between extracellular and intracellular spaces to control diverse cellular functions. TM4SF5 influences the epithelial-mesenchymal transition (EMT), aberrant multilayer cellular growth, drug resistance, enhanced migration and invasion, circulation through the bloodstream, tumor-initiation property, metastasis, and muscle development in zebrafish. Here, current data on TM4SF5's roles in the development of fibrotic phenotypes are reviewed. TM4SF5 is induced by transforming growth factor *β*1 (TGF*β*1) signaling via a collaboration with epidermal growth factor receptor (EGFR) activation. TM4SF5, by itself or in concert with other receptors, transduces signals intracellularly. In hepatocytes, TM4SF5 expression regulates cell cycle progression, migration, and expression of extracellular matrix components. In CCl_4_-treated mice, TM4SF5, *α*-smooth muscle actin (*α*-SMA), and collagen I expression are observed together along the fibrotic septa regions of the liver. These fibrotic phenotypes are diminished by anti-TM4SF5 reagents, such as a specific small compound [TSAHC, 4′-(*p*-toluenesulfonylamido)-4-hydroxychalcone] or a chimeric antibody. This review discusses the antifibrotic strategies that target TM4SF5 and its associated protein networks that regulate the intracellular signaling necessary for fibrotic functions of hepatocytes.

## 1. Introduction

Membrane proteins and receptors can be enriched at unique and specific regions on a cell's surface. These enriched sites include focal adhesions, lipid rafts, caveolae, and tetraspanin-enriched microdomains (TERMs). Tetraspanins in TERMs can form massive protein-protein complexes that transduce extracellular signals to intracellular signaling pathways [1]. These membrane architectures would have unique protein compositions that can result from differential trafficking; complex formation for activation of special intracellular signaling pathways by these complexes occurs in a spatiotemporal manner [2, 3]. Chronic injury and inflammation in the liver results in diverse environmental stimuli which can cause hepatocytes to eventually develop fibrotic phenotypes. Fibrotic phenotypes are regulated by diverse soluble inflammatory factors including cytokines, chemokines, and growth factors, in addition to extracellular matrix (ECM). Membrane proteins and receptors that respond to the extracellular cues can be involved in the development of the fibrotic phenotypes. Furthermore, specific strategies targeting membrane proteins could result in promising antifibrotic reagents that maybe clinically valuable. Because it is a member of the transmembrane 4 L six family similar to the tetraspanins (TM4SFs), TM4SF5's role in the development of fibrotic phenotypes is discussed in this review.

## 2. TM4SF5-Enriched Microdomain (T_5_ERM) and Cell Migration Property

The transmembrane 4 L six family is comprised of TM4SF5, TM4SF1 (L6, L6-Ag), TM4SF4 (IL-TIMP), TM4SF18 (L6D), TM4SF19, and TM4SF20 [2, 4]. Members of this family show a membrane topology similar to those of genuine tetraspanins (TM4SFs). They have four transmembrane (TM) domains, two short cytosolic N- and C-terminal tails, and two extracellular loops (SEL and LEL for short extracellular loop 1 and long extracellular loop 2, resp.). Slight differences in the LELs are observed between members. Members of the transmembrane 4 L six family have relatively variable LELs, whereas the LELs of genuine tetraspanins have a variable region and a conserved region with a CCG moiety and four conserved cysteine residues [3, 5]. There have been reported 33 mammalian tetraspanins that are generally at 20~30 kDa [1].

Tetraspanin complexes together with other receptors at TERMs play dynamic roles in regulating cellular functions. Recently, we have shown that TM4SF5 forms a protein complex with EGFR and integrin *α*5 at the leading membrane edges [6]; dynamic intra-TERM protein associations and translocations during cell migration depend on interactions mong TM4SF5, EGFR, and integrin *α*5 at the leading membrane edges of migratory cells to steer migration. These interactions transduce differential synergistic signals in 2-dimensional (2D) and 3-dimensional (3D) culture systems and determine the directional migration. Furthermore, the association of TM4SF5 with EGFR and integrin *α*5 is dynamically coordinated; their affinities for each other depend on intracellular cholesterol levels and posttranslational modification (PTM) level. TM4SF5 can be *N*-glycosylated and palmitoylated. Upon M*β*CD treatment to deplete cellular cholesterol, only integrin *α*5 remains alone at the leading edges, whereas TM4SF5 and EGFR translocate to “internal T_5_ERMs,” where they cover an area of plasma membrane surface distal to the membrane boundaries, depending on TM4SF5 PTMs. The interaction between TM4SF5 and EGFR is stronger and occurs at the leading area of migratory cells. In contrast, TM4SF5's association with integrin *α*5 is less strong and is distributed evenly in migratory cells. These coordinated TM4SF5 interactions with EGFR and integrin *α*5 depend on cues from both intracellular and extracellular spaces. These interactions control the directional migration properties such as directionality and speed in 2D and 3D cells and spheroid culture systems. Thus, the differential association and localization of TM4SF5 with EGFR and/or integrin *α*5 fine-tune signaling activities that regulate dynamic cellular and biochemical processes in the leading edge of migratory cells. The dynamics of these coordinated protein associations within T_5_ERMs, which occur proximal to or distal from the leading membrane edges, depend on cellular cholesterol levels and the PTMs of TM4SF5.

In addition to EGFR and integrin *α*5, TM4SF5 binds to CD44. This interaction promotes drug resistance and circulating and tumor-initiating properties [7]. The interaction between TM4SF5 and CD44 involves the LEL of TM4SF5 where *N*-glycosylation occurs; a *N*-glycosylation-deficient mutant and treatment with 4′-(*p*-toluenesulfonylamido)-4-hydroxychalcone (TSAHC), a small compound that inhibits TM4SF5 [8], abolishes the interaction between TM4SF5 and CD44, which results in no such properties and no spheroid growth in 3D aqueous culturing conditions [7]. Furthermore, TM4SF5 interacts with CD151, but not with CD63, to regulate the migration and invasion capacity of hepatocytes [9]. Overexpression of promigratory TM4SF5 results in an internalization of CD63 to late lysosomal membranes and prevents the inhibitory effect of CD63 on migration by presumably removing it from the cell surface [9]. Therefore, TM4SF5 expression can modulate localizations of other membrane receptors like CD63 [9], EGFR, or integrin *α*5 [6], although its effects on CD44 localization have not been examined. Finally, the interaction among TM4SF5, integrin *α*2 [10], and *α*5 can modulate cell migration, invasion, and vascular endothelial growth factor (VEGF) expression. This contributes to the angiogenic capacity [11].

Interestingly, T_5_ERM formation also appears to be dependent on cellular cholesterol levels and TM4SF5 PTMs, which are important for the protein-protein interactions. Composition of the T_5_ERMs may also be dependent on cholesterol levels. In normal migratory conditions, TM4SF5 colocalizes together with EGFR and integrin *α*5 at the very tip of the leading edge membranes (i.e., boundary T_5_ERMs); upon depletion of cholesterol, TM4SF5 and EGFR translocate together to the “internal T_5_ERMs” while integrin *α*5 remains at the membrane edges presumably at focal adhesions. Further, when the cell fractionation is performed with Brij97 detergent, TM4SF5 is recovered from a different fraction than the one containing lipid rafts that are enriched with caveolin 1. Collectively, these data suggest that T_5_ERMs are different from lipid rafts and focal adhesions and that they are located either at membrane boundaries or at internal membranes based on dynamic binding partner exchanges. This information can be used to develop approaches to manipulate cellular migration processes.

## 3. TM4SF5 Only-Mediated Signal Transduction

Although TM4SF5 binds to many different membrane receptors including CD151, growth factor receptors, and integrins [6], TM4SF5 itself can trigger intracellular focal adhesion kinase (FAK) [12] and c-Src-dependent signaling [13]. Direct interaction of the intracellular loop (ICL) or the cytosolic C-terminal tail of TM4SF5 with FAK or c-Src modulates directional migration and invasion, respectively. Interaction of the ICL of TM4SF5 with the F1 lobe in the four-point-one, ezrin, radixin, moesin (FERM) domain of FAK releases the inhibitory intramolecular interaction between the FERM domain (F2 lobe) and the kinase domain (C lobe centered on Phe562) leading to transautophosphorylation of the Tyr 397, which is buried by the intramolecular interaction in FAK [12]. Thus, TM4SF5 expression in hepatocytes can promote phosphorylation of Tyr 397 in FAK upon cell adhesion. TM4SF5-mediated FAK phosphorylation is possible even without the involvement of integrin engagement to ECM components. When cells are replated onto inert materials of poly-L-lysines or when cells precoated with anti-integrin *β*1 antibodies are replated onto fibronectin, TM4SF5-mediated FAK phosphorylation is higher than when cells are kept in suspension. These data indicate that TM4SF5 itself mediates FAK activation via a direct interaction between TM4SF5 and FAK. This TM4SF5-mediated activation process appears to involve a conformational release in FAK upon cellular adhesion.

In addition, TM4SF5-positive cancerous hepatocytes also show phosphorylation at Tyr 705 of STAT3, presumably independent of IL-6 treatment [14]. Molecule upstream of STAT3 can include c-Src [15] and JAKs [16]. Therefore, the direct interaction of the C-terminal tail of TM4SF5 with c-Src can lead to STAT3 activation. IL-6-mediated STAT3 activation appears to inhibit cell spreading, migration, and invasion, whereas TM4SF5-mediated STAT3 activation may lead to promotion of the effects, based on the fact that TM4SF5-positive cancerous hepatocytes express less IL-6 [14]. Recently, we also observed that TM4SF5-mediated STAT3 signaling activity contributes to the expression of ECMs (Ryu J and Lee JW, unpublished data). Therefore, TM4SF5 itself can transduce signals for intracellular FAK, c-Src, and STAT3 pathways to regulate cell migration and possibly ECM production. These data indicate that TM4SF5 is important for tyrosine phosphorylation-related pathways.

Furthermore, the structural aspect or relay from the *N*-glycosylated LEL to the cytosolic C-terminus through the 4th transmembrane domain appears to be critical for TM4SF5-mediated signaling activation of FAK and c-Src [17]. Cysteines proximal to transmembrane domains are involved in palmitoylation that is critical for the association of TM4SF5 with tetraspanins and other receptors [6]. Meanwhile, TM4SF1 and TM4SF4, which are also transmembrane 4 L six family members, differ from TM4SF5 in the ability to activate FAK and/or c-Src to regulate migration [17]. Although TM4SF5 alone can modulate signaling pathways, it can also collaborate with other membrane proteins and receptors to control intracellular signaling pathways.

## 4. Signaling Activity Modulated by TM4SF5 and Its Binding Partners

T_5_ERMs can have many different components, and various protein-protein interactions among diverse membrane proteins and receptors can occur [6]. Formation of protein complexes among TM4SF5, EGFR, and integrin *α*5 leads to differentially coordinated signaling activities depending on the PTMs of TM4SF5, which are discussed above. Wild-type TM4SF5 coordinates adhesion- and EGFR-mediated signaling pathways. A palmitoylation-deficient TM4SF5 mutant fails to coordinate adhesion-mediated FAK activity upon EGF treatment, presumably because there is a synergistic relationship between adhesion- and EGFR-mediated signaling activities in wild-type cells that does not occur in cells expressing the palmitoylation-deficient mutant of TM4SF5.

Although TM4SF5 does not belong to the genuine tetraspanin family [4], TM4SF5 is a member of the transmembrane 4 L six family member and can form TERMs with other receptors and tetraspanins. These TERMs play important roles in the regulation of metastatic potentials. TM4SF5 interacts with integrins *α*2, *β*1 [18], and *α*5 [11]; EGFR [19]; TGF*β*R [20]; and CD44 [7]. In addition to these binding partners, TM4SF5 binds the tumorigenic tetraspanin CD151, but not tumor-suppressive tetraspanin CD63 [9]. These data support a role for TM4SF5 in tumor progression as a tetraspanin-like molecule at T_5_ERMs. However, a binding protein or receptor of TM4SF5 that plays a role in the development of fibrotic phenotypes has not yet been identified, although TGF*β*R, which is important for TM4SF5 expression during CCl_4_-induced fibrosis, has been shown to interact with TM4SF5 [20]. Because fibrotic liver phenotypes may result from diverse inflammatory cytokines and chemokines produced following chronic injury and inflammation and complex gene regulation events, TM4SF5 may also be involved in the trafficking and stabilization of different membrane receptors via complex formation. Furthermore, it is currently not known whether fibrotic phenotypes are caused by unbalanced levels of cell death, cell survival, or regeneration following chronic hepatocyte injury. These processes may involve integrins and growth factor receptors that can bind to TM4SF5.

## 5. Regulation of TM4SF5-Mediated Fibrotic Phenotypes

Because TM4SF5 expression suppresses E-cadherin, which leads to the epithelial-mesenchymal transition (EMT) process [21], it is involved in organ fibrosis. Additionally, because hepatocellular carcinoma can arise from liver fibrosis/cirrhosis [22], it is reasonable to hypothesize that TM4SF5 overexpression in liver cancer tissues [23] could be involved in liver fibrosis. Consistent with this idea, TM4SF5 expression increases in a CCl_4_-induced mouse liver with fibrotic phenotypes. In this model, *α*-SMA expression and collagen I deposition [24] are regulated by TGF*β*1- and EGFR-mediated signaling pathways [20]. The multifunctional cytokine TGF*β*1 is involved in fibrosis and tumorigenesis via the induction of EMT [25]. TGF*β*1 induces TM4SF5 expression [20]. Consequently, TM4SF5-expressing hepatocytes acquire mesenchymal features via a cross talk between activated SMADs and EGFR/ERK signaling pathways. Thus, TM4SF5-mediated fibrotic effects could be suppressed by inhibiting TGF*β*1-mediated R-SMAD activation, EGFR activation, or TM4SF5 function [20].

TM4SF5 expression correlates with *α*-SMA expression in the liver of CCl_4_-treated mice, possibly indicating an EMT process and/or hepatic stellate cell (HSC) activation [26], although an EMT process during liver fibrosis has not been confirmed in vivo. In this CCl_4_-induced model of liver fibrosis, multifunctional TGF*β*1 can mediate the EMT and/or activation of HSCs leading to active myofibroblasts, which are marked by enhanced *α*-SMA expression. TM4SF5, collagen I, and *α*-SMA expressions along the fibrotic septa are observed in the livers of CCl_4_-treated mice [27, 28]. Further, we observed differences in the expression of ECM components (i.e., collagen I, laminin, and fibronectin) when comparing albumin-positive hepatocytes and *α*-SMA-positive myofibroblasts in the livers of CCl_4_-treated mice (Ryu J and Lee JW, unpublished observations). Thus, TM4SF5 may be involved in the activation of HSCs or myofibroblasts, which eventually produces and deposits the collagen I involved in fibrosis [24]. Furthermore, TM4SF5 in hepatocytes can contribute to the activation of myofibroblasts and/or the induction of ECMs. These liver injury and fibrotic phenotypes, including ECM deposition, *α*-SMA expression, and fibrotic septa formation in the livers of CCl_4_-treated mice, are attenuated by the administration of the anti-TM4SF5 reagent, TSAHC [8, 24], or a chimeric anti-TM4SF5 antibody (Lee JW and Kim S, unpublished observations). Therefore, TM4SF5 is an important player during TGF*β*1 and soluble factor-mediated activation of liver cells, because it regulates ECM production and contributes to the development of liver fibrosis.

## 6. Concluding Remarks

*TM4SF5* was identified as a tumor-associated antigen, similar to L6 which is a lung tumor-associated antigen, via a large-scale screen for differentially expressed genes in pancreatic cancer in 1998 [29]. Since then, TM4SF5 has been investigated for its roles in diverse cellular functions by mostly us and others. TM4SF5 regulates diverse cellular functions and behaviors, including actin organization, focal adhesion turnover, cell proliferation, EMT, migration, invasion, drug resistance, and circulating tumor cell and tumor initiating cell properties. Importantly, TM4SF5 functionally contributes to liver diseases including metabolic disorders, inflammation, and fibrosis of the liver. Thus, it is likely that TM4SF5 chronically drives different steps of liver diseases, eventually leading to hepatic cancer and metastasis.

During these TM4SF5-mediated processes that contribute to liver fibrosis and cancer development, it seems that this tetraspanin, TM4SF5, plays important roles in the regulation of cellular functions related to the diseases via signal transduction. TM4SF5 can work alone or in collaboration with other proteins to regulate signaling events. As a component of T_5_ERMs, TM4SF5 modulates signaling in a spatiotemporal manner by altering the localization, trafficking, stability, and binding affinity and partners. Therefore, TM4SF5 can help to regulate different and important cellular functions.

Here, we have discussed the effects of TM4SF5 expression on hepatocytes during liver fibrosis. Further, we have studied the connection between the ECM and TM4SF5 in hepatocytes, which will soon become public. Because hepatocytes are a major liver cell type and also easily and chronically injured, they are important mediator of liver diseases. In the future, we can address the roles of TM4SF5 in hepatocytes and how this protein contributes to liver disease for the purposes of development of antifibrotic reagents that are therapeutically promising.

## Abbreviations

ECM: Extracellular matrix
EMT: Epithelial-mesenchymal transition
FAK: Focal adhesion kinase
ICL: Intracellular loop
LEL: Long extracellular loop 2
PTM: Posttranslational modification
SEL: Short extracellular loop 1
STAT3: Signal transducer and activator of transcription 3
T_5_ERM: TM4SF5-enriched microdomain
TERM: Tetraspanin-enriched microdomain
TSAHC: 4′-(*p*-Toluenesulfonylamido)-4-hydroxychalcone
*α*-SMA: *α*-Smooth muscle actin.
